# Hypermobility spectrum disorders: A review

**DOI:** 10.2478/rir-2023-0010

**Published:** 2023-07-22

**Authors:** Matthew B Carroll

**Affiliations:** Rheumatology, Singing River Health System, 3603 Bienville Blvd, Ocean Springs, MS 39564, USA

**Keywords:** joint hypermobility, generalized joint hypermobility, joint hypermobility syndrome, benign joint hypermobility syndrome, hyper-mobility spectrum disorder, hypermobility Ehlers-Danlos syndrome

## Abstract

It remains a clinical challenge identifying when joint hypermobility (JH) is responsible for pain. Previous nomenclature utilized terms such as (benign) joint hypermobility syndrome (JHS) but this was updated in 2017 as advances in genetics provide a basis for nearly all variants of Ehlers-Danlos syndrome (EDS) with the exception of hypermobile EDS (hEDS). New terminology describes hypermobility spectrum disorders (HSDs) as the updated term for JHS. Diagnosis of a subtype of HSDs should be considered in patients who have JH coupled with the presence of secondary musculo-skeletal manifestations (trauma, chronic pain, disturbed proprioception, and other manifestations) and at the exclusion of hEDS. Extra-articular manifestations are common. Treatment relies on management strategies for other chronic pain syndromes with a multidisciplinary approach likely optimal. Lifestyle modifications focus on weight loss and exercise. Physical therapy helps strengthen periarticular muscles, improving mobility. Pharmacologic therapies focus on judicious use of non-steroidal anti-inflammatory drugs and acetaminophen. Serotonin and norepinephrine reuptake inhibitor may help widespread pain. Avoidance of opioids remains prudent. The purpose of this review is to provide clinicians the rationale for the update in nomenclature, understand the musculoskeletal and extra-articular manifestations of the subtypes of HSDs, considerations when making the diagnosis, and treatment.

## Introduction

The relationship between joint hypermobility (JH) and pain was described in the mid-to-late 1960s.^[1]^ Initially pain related to join hypermobility was presumed isolated to the musculoskeletal system and affect otherwise healthy patients. It was also considered distinct from heritable disorders of connective tissue such as Ehlers-Danlos syndrome (EDS). Up until 2017 this clinical syndrome was called the joint hyper-mobility syndrome (JHS) or benign joint hypermobility syndrome (BJHS).^[2,3]^ Over the last two decades, greater clinical awareness of the relationship between musculoskeletal pain and JH, coupled with identification of novel genes related to clinical variants of EDS, highlighted difficulty distinguishing patients with BJHS from those with heritable disorders of connective tissue, the most frequent being EDS, hypermobility type (type III prior to 2017) (EDS-HT).^[4]^

In 2017 an update in nosology was published by the Ehlers-Danlos Society, replacing clinically overlapping and occasionally confusing terms of BJHS and EDS-HT. This work was the culmination of years of expert opinion^[5]^ and a familial study demonstrating co-segregation of these two disorders in pedigrees with multiple affected members fitting criteria for both.^[6]^ Despite genetic testing more readily available to assist in the diagnosis of various EDS subtypes, the hypermobility EDS variant still has no known genetic marker.^[4]^ Thus, within the EDS nomenclature the term hypermobility EDS (hEDS) was introduced and a new set of diagnostic criteria provided.^[7]^ For other patients who have symptomatic JH, do not have a known subtype of EDS, and do not meet diagnostic criteria for hEDS, they now have a subtype within the hypermobility spectrum disorders (HSDs).^[4]^

Historically the diagnosis of symptomatic JH, be it attributable to BJHS, EDS-HT, or now to hEDS or a subtype of HSDs, has been challenging. With a prevalence approaching 4% of the general populations, underdiagnosis has been common.^[8]^ The lack of clinician familiarity and experience with the disorders, a highly variable clinical presentation, and absence of a confirmatory test, lead to continued under recognition of these disorders.^[8]^ Adding to the challenge of identifying patients with HSDs is the high rate of extra-articular manifestations. The purpose of this review is to update clinicians across the spectrum of healthcare about the concept and clinical impact of HSDs, the reason for and significance of the update in nomenclature, the impact that these disorders have with regard to causing chronic musculoskeletal pain, the clinical manifestations observed beyond the joints, and how to identify, diagnose, and treat patients with HSDs. This review will specifically focus on patients 18 years of age or older.

## Defining & Identifying Joint Hypermobility

At the core of all HSDs, along with other heritable disorders of connective tissue, is the presence of JH. JH (also double jointed, joint laxity, or joint hyperlaxity) describes the capability of a joint (or group of joints) to move passively or actively beyond normal limits along physiologic axes.^[4]^ The term JH is not diagnostic but instead a description of a clinically identifiable finding, either reported on history or observed on physical examination.

If clinical suspicion exists that a patient has JH, several confirmatory assessments are available. A screening questionnaire is available, known as the 5-Part Questionnaire (5PQ).^[9,10]^ It consists of 5 questions about JH, as shown in Table 1. Generalized JH (GJH) is considered present if a positive response is obtained to at least 2 of the questions.^[10]^ Reported sensitivity and specificity for the 5PQ is 84% and 85% respectively.^[8,10]^ Physical examination may also identify JH. This can be assessed using a goniometer, measuring observed maximal range of motion (ROM) and comparing results appropriate for the age and gender of the patient. Another way to identify JH is to use the Beighton scoring system. This technique utilizes 9 quick, easy to perform examination maneuvers and are listed in Table 2.^[11]^ The presence of an observed finding is 1 point and scores range from 0 to 9. For adults, a cut-point of at least 4 out of 9 points is suspicious for GJH.^[3,9]^ Measurements obtained with the goniometer and BS may be influenced by age, gender, ethnicity, and physical fitness.^[5,11]^

**Table 1:** The 5-Part Questionnaire (5PQ). Yes/No questions to ask patients with suspected JH

5-Part Questionnaire (5PQ)	Answer
(1) Do you consider yourself double-jointed?	Yes/No
(2) Can you now (or could you ever) place your hands flat on the floor without bending your knees?	Yes/No
(3) Can you now (or could you ever) bend your thumb to touch your forearm?	Yes/No
(4) As a child, did you amuse your friends by contorting your body into strange shapes or could you do the splits?	Yes/No
(5) As a child or teenager, did your shoulder or kneecap dislocate on more than one occasion?	Yes/No

Positive responses to at least 2 of these questions have reported sensitivity of 84% and specificity of 85% for the previously described joint hypermobility syndrome (JHS), as characterized by the Brighton Criteria for JHS. Applicable to the identification of generalized joint hypermobility (GJH).

**Table 2:** Beighton score (BS) for suspected JH

Items	Right	Left
Passively dorsiflex the 5^th^ metacarpophalangeal joint to ≥ 90°	1	1
Oppose the thumb to the volar aspect of the ipsilateral forearm	1	1
Hyperextend the elbow to ≥ 10°	1	1
Hyperextend the knee to ≥ 10°	1	1
Place hands flat on the floor without bending the knees (“palm the floor”)	1	
TOTAL SCORE (maximum of 9 points)		

When JH is observed at one or a few types of joints, this is defined as localized JH (LJH).^[4]^ LJH typically affects a single small or large joint but may be bilateral, such as observed bilateral genu recurvatum. When multiple joints (5 or more) are involved, the term GJH applies, and should involved all limbs and the axial skeleton.^[4]^ The clinical decision making regarding whether or not a patient has GJH is not necessarily as straightforward as it would seem, especially when joint ROM changes over time and there is no standardized approach that remains valid and reproducible under diverse clinical circumstances. Other subtypes of JH may also exist based on clinical practice and the literature.^[4]^ Peripheral JH (PJH) is a potential form of JH appreciable in the hands and/or feet. It is distinct from LJH as it involves the four limbs but distinct from GJH as it spares the large joints and axial skeleton.^[4]^ It may be clue to vascular EDS.^[4]^ Historical JH is self-reported (through the 5PQ) GJH but with a negative Beighton score (BS).

## Nomenclature

There are currently four different subtypes in the spectrum of HSDs. They share JH with other conditions, notably EDS, and these subtypes do not appear to be Mendelian disorders.^[4]^ While JH is asymptomatic, there exist musculoskeletal symptoms and complications that may reasonably be considered secondary manifestations of JH. These secondary musculo-skeletal manifestations are listed in Table 3 and include trauma (micro- or macro-), chronic pain, disturbed proprioception (reduced proprioception with muscle weakness), and other musculoskeletal traits (pes planus, valgus deformities, scoliosis, and others).^[4]^

**Table 3:** Secondary musculoskeletal manifestations of JH^[4]^

Trauma	Macrotrauma-Dislocations, subluxations, or any form of damage to muscles, ligaments, tendons, synovium, or cartilage the result of excessive joint movement along non-physiologic axes Microtrauma-Subtle or silent injury perhaps not perceived by the individual or clinician as it occurs. Over time likely predisposes to recurrent/persistent pain and early joint degeneration
Chronic Pain	Occasional/recurrent pain in affected joint (immediate consequence) Development of hyperalgesia as a possible form of pain sensitization. Higher rate of small fiber neuropathy in adults with common EDS subtypes leads to speculation regarding direct relationship between impaired connective tissue function and abnormal pain processing.
Disturbed proprioception	Reduced proprioception in selected joints with muscle weakness fuels. These influence each other and likely generate a “vicious cycle” of increasing limitation of activities
Other musculoskeletal traits	Musculoskeletal physical traits which may result from laxity in soft tissues surrounding joint and mechanical forces during growth and development Pes planus Valgus deformity of elbows, hind feet and halluces, Scoliosis (not congenital, mild to moderate degree ≥ 7° using the Bunnel scoliometer Accentuated dorsal kyphosis and lumbar lordosis, and Deformational plagiocephaly

The four currently described subtypes of HSDs, in line with the aforementioned subtypes of JH, are:^[4]^

1. Generalized (joint) HSD (G-HSD): GJH objectively assessed utilizing a clinical score such as the Beighton score plus one or more secondary musculoskeletal manifestations.
  a. Differential diagnosis for these patients, in the context of the pattern and severity of their symptoms and clinical findings, could be due to hEDS.
2. Peripheral (joint) HSD (P-HSD): JH limited to hands and feet plus one or more secondary musculoskeletal manifestations.
3. Localized (joint) HSD (L-HSD): JH at single joints or group of joints plus one or more secondary musculoskeletal manifestations regionally related to the hypermobile joint(s).
4. Historical (joint) HSD (H-HSD): self-reported (historical) GJH by the 5PQ with a negative BS plus one or more secondary musculoskeletal manifestations.

Table 4 summarizes the different subtypes of HSDs.^[4]^ The updated 2017 terminology substitutes all previous terms used to define patients with JH but without a molecularly proven condition. Terms now outdated are EDS type III, EDS-HT, hypermobility syndrome, JHS, and BJHS.^[4]^

**Table 4:** Distinguishing HSDs^[6]^

Phenotype	Beighton Score^*^	Musculoskeletal Involvement
G-HSD	Positive	Present
P-HSD	Usually negative	Present
L-HSD	Negative	Present
H-HSD^+^	Negative	Present
hEDS	Positive	Possible

^*^ A positive Beighton Score is considered a value ≥ 4 (out of 9).

^+^ : In H-HSD the 5PQ is positive indicating the historical presence of JH.

## Epidemiology

Understanding the epidemiology of HSDs has been hampered by a variety of factors. These include the updated nosology but also whether JH, GJH, or BJHS were being studied, the instrument to measure JH (for example, 5PQ *vs*. BS), and the specific group of people being studied. With the elimination of the term BJHS in 2017, figures are now used to describe the combined prevalence of HSD and hEDS.^[12]^ The combined prevalence of HSDs and hEDS is 1 in 600 to 1 in 900. Expert opinion is that HSDs are common and that hEDS is likely to be common.^[12]^ The current prevalence figures are also likely underestimates because many people with EDS or HSDs experience a delay in diagnosis.^[12]^ Prevalence of the BJHS was about 3%-4% of the general population, affecting 3.3% of women and 0.6% of men.^[8,13]^ This data was obtained from a cross-sectional population survey in the United Kingdom. Twenty-eight percent returned a survey with complete data. Around 18.3% classified as being hypermobile and 16.3% having chronic widespread pain. Those with JH were more likely to report chronic widespread pain (18.5% *vs*. 15.8%; P < 0.001), and hypermobile participants were 40% more likely to report the most severe pain.^[13]^ Another study utilized a nationwide linked electronic cohort and nested case-control design.^[14]^ Described using BJHS, the study identified 6021 individuals, of whom 30% were men and 70% were women.^[14]^ A prevalence of 194.2 per 100,000 in 2016/2017 was noted with a sizeable gender difference of 8.5 years in the mean age at the time of diagnosis.

Incidence and prevalence estimates for GJH vary. The prevalence of GJH in children and young adults under the age of 19 is 32.5% for girls and 18.1% for boys, with a prevalence of JH around the world at 34.1% in this study.^[15]^ Studies of “college students” (typical ages between 18 and 24) report a GJH prevalence between 11%-12.5%^[16,17]^ and other between 12.5%-26%.^[2,18,19]^ Among professional dancers, the prevalence ranges between 64.9% and 72%.^[20,21]^ GJH also appears to be more likely in younger females and less likely in older males.^[15]^ A large community based study which examined the frequency of GJH and how it was related to osteoarthritis (OA) by ethnicity (African American *vs*. Caucasian) reviewed data collected in the Johnston County OA project database from 2003-2010.^[22]^ From this group, 8% of Caucasians were hypermobile *vs*. 5% of African Americans, a statistically significant difference.^[22]^

## Pathophysiology

The specific underlying causes and mechanisms responsible for the musculoskeletal pain reported by patients in HSDs require further extensive investigation. With the evolution of the understanding of the molecular changes responsible for most variants of EDS as well as the nosology update in 2017, the genetic backgrounds of both HSDs and hEDS are currently unknown but seem to have a weak autosomal dominant pattern.^[4,23]^ For readers interested in the currently known genetic basis of various EDS variants, the author recommends the summary provided by Malfait *et al*.^[23]^ published in 2017. For environmental factors, current theories emphasize localized biomechanical overloading and chronic soft tissue injury due to joint laxity and instability.^[2]^ This likely leads to soft tissue injury manifesting as localized joint arthralgias (nociceptive pain) and in some, over time, diffuse musculoskeletal pain (central sensitization).^[2,8,24,25]^ Patients with HSDs appear to lack proprioceptive acuity, which may increase risk of injury due to difficulty judging joint position.^[26]^ A 2010 meta-analysis of 18 studies concluded that there is an increased risk of knee injury but not of the ankle.^[27]^ Compounding the effects of altered loading on the joint is an observed decrease in muscle mass and muscle strength. Increased tendon laxity leads to difficulty transmitting power produced by muscles.^[28,29]^ Two studies comparing patients with asymptomatic GJH with BJHS found patients with BJHS tended toward decreased joint momenta in the lower extremities, necessitating a greater force to maintain equilibrium.^[30,31]^ Of note, these conclusions have been refuted by other studies, with a systematic review questioning the clinical relevance of differences in gait and the effect that it has in the musculoskeletal pain of BJHS.^[32]^

Beyond biomechanical factors, patients with HSDs may have neurologic perturbations that contribute to diffuse musculoskeletal pain. Generalized hyperalgesia is common and believed to be mediated by central sensitization. Fear of provoking pain and sustaining injury potentially leads to decreased levels of physical activity, hastening deconditioning and exercise intolerance.^[8,33]^ This has been found in high performance athletes with GJH,^[11]^ and it has been speculated that this serves as a compensatory mechanism to prevent joint instability. Cross-sectional studies suggest that chronic trauma reduces the pain threshold,^[8]^ and hyperalgesia may serve as a compensatory mechanism to minimize joint instability.^[34]^ Knowledge about the pathophysiologic mechanisms that link HSDs to extra-articular manifestations is even less clear. Previously described in BJHS, higher rates of anxiety and depression may be due to pain-related fear and fear avoidance, but studies are scarce to clarify this relationship.^[8,35,36]^ Even less is understood about the autonomic dysfunction patients may experience potentially due to connective tissue abnormalities.^[8,36]^

## Clinical Manifestations

Symptoms may have a highly variable presentation, with patients likely coming to the attention of the medical establishment because of their musculoskeletal symptoms. With a possible onset at any age, even as late as the 6^th^ or 7^th^ decade of life, the onset of pain typically starts in the early to mid-teenage years. Pain may be reported as a dull to moderate ache, limited to activities or constant in nature. Weight bearing joints may be more symptomatic and/or interfere with the ability to perform activities of daily living.^[8]^ The joint pain may be worse with physical activity or repetitive use, potentially improving with decreasing the intensity of the activity. Prolonged morning stiffness lasting more than 30 min is unusual,^[35]^ although with chronic widespread pain prolonged morning stiffness may last longer.

The review of systems may capture information about the extra-articular manifestations of HSDs. Patients may have mental health, nervous system, and digestive system issues with a higher odds of receiving a prescription in other disease categories (such as gastrointestinal and cardiovascular drugs) within 12 months before and after the diagnosis.^[14]^ The strongest associations of HSDs are with orthostasis, a variety of functional gastrointestinal disorders, and pelvic and bladder dysfunction.^[4]^ Table 5,^[37, 38, 39, 40, 41, 42, 43, 44, 45, 46, 47, 48, 49, 50, 51, 52, 53]^ though not exhaustive, summarizes the growing list of extra-articular manifestations associated with HSDs.

**Table 5:** Extra-articular manifestations of the subtypes of HSDs to screen for during a review of systems^[4,8,37]^

ROBUST ASSOCIATIONS			
Issue	Potential causes	History, symptoms, or clinical findings	Select Additional References
Mood	Unclear	Fear Emotional distress Anxiety Depression Panic attacks Somatoform disorder	[41,43,44]
Fatigue (up to 84%)	Unclear, but muscle weakness may be one of multiple etiologies	Sleep disturbances Nonrestorative sleep Concentration difficulties Nocturnal musculoskeletal pain	[44,45]
Functional gastrointestinal disorders	Ligamentous laxity and abnormal connective tissue Altered gut-brain neurologic interactions Visceral hypersensitivity Altered vascular compliance	Nausea Vomiting Diarrhea Constipation Early satiety Bloating Rumination Reflux/heartburn	[38,40,42]
Dysautonomia (up to 75%)	Increased sympathetic tone Decreased sympathetic reactivity to stimuli Increased vascular distensibility Comorbid depression Low-level pain-induced sympathetic arousal	Orthostatic intolerance/hypotension (dizziness with postural changes) Secretomotor complaints (abnormal sweating, sicca-like symptoms) Raynaud’s phenomenon History of Postural Orthostatic Tachycardia Syndrome (POTS)	[39,49,51]
Pelvic Girdle Pain	Altered biomechanics and transmission of forces through spine Asymmetry of pelvic ligaments > 5 mm	Pain Dyspareunia Organ prolapse Long latent phases of labor with rapid progression Association with polycystic ovaries, fibroids, endometriosis	[46,47]
**POTENTIAL ASSOCIATIONS**			

Mitral valve prolapse			[48]
Aortic root dilatation			[48]
Mast cell activation syndrome			[49,50]
Autism			[52,53]

Family history is also important to obtain. It is possible that a family history of JH or GJH will be reported by a patient using a term such as “double jointed”. Whether or not these family members have pain will vary, but some family members might have been diagnosed with BJHS classical EDS, vascular EDS, and hEDS have an autosomal dominant pattern of inheritance,^[23]^ although sometimes with variable expression.^[4]^

Physical examination should utilize the BS, focus on a thorough musculoskeletal exam, and assess cutaneous findings that might point to a heritable disorder of connective tissue. Joints should be examined for tenderness, swelling, redness, and deformities in addition to assessing ROM.^[8]^ Overt synovitis is unusual, but some patients may have small effusions as a consequence of meniscal or cartilaginous irritation. Some patients may have scoliosis, lordosis, pes planus, valgus deformities of the knees, or easy joint subluxation. Musculoskeletal findings that might be a clue that the patient has a heritable disorder of connective tissue would be arachnodactyly or an arm span-to-height ratio ≥ 1.05.^[23]^ Cutaneous findings inconsistent with HSDs are unusually soft or velvety skin, mild skin hyperextensibility, piezogenic papules of the heel, and atrophic scarring.^[23]^ The presence of mitral valve prolapse (MVP) or aortic root dilatation on echocardiograph may again point to a diagnosis other than a subtype of HSDs.

## Diagnosis

For diagnosis, JH should be identified at some point during the evaluation of the patient coupled with the presence of secondary musculoskeletal manifestations, and with the exclusion of hEDS. Conceptually, asymptomatic JH, the HSDs, and hEDS fall on a spectrum ranging from isolated JH to hEDS.^[4]^ Patients may potentially transition through various HSDs as their symptoms evolve.^[4]^ It is paramount to exclude the possibility that a patient has hEDS. The process of excluding hEDS make require several visits and expert evaluation given the need for detailed history taking and observing subtle clinical findings. The author recommends using the 2017 International Classification of the Ehlers-Danlos Syndromes by Malfait *et al*.^[23]^ for the criteria to diagnose hEDS.

## Management

A multidisciplinary approach is presumed to be the best way to globally address symptoms. The focus should address the cause of the pain (for example, joint dislocation, muscle imbalance with spasm, sprain/strain), reducing pain when possible, and maximizing functional capacity and quality of life.^[2]^ Treatment of dysautonomia in patients with HSD may improve both fatigue and pain.^[54]^ The majority of patient with a subtype of HSD may reasonably be managed at a primary care level, but access to physical therapists as well as mental health specialists skilled in biofeedback and cognitive behavioral therapy may also provide substantial supportive care.^[2,55]^ Plausibly other specialists such as rheumatologists, pain managers, sport medicine specialists, or orthopedic surgeons may be needed. Extra-articular manifestations may also require subspecialist evaluation and management, for example, dysautonomia and postural orthostatic tachycardia syndrome (POTS) managed by a cardiologist.

Two limitations exist with the management strategy noted. First, specialists with familiarity assisting the management of a subtype of HSD may not be readily accessible. A survey distributed to rheumatology occupational and physical therapists in Scotland found that they had expertise in the management of HSD/hEDS patients however, the expertise was concentrated in higher levels of care, making it a challenge for patients managed at a primary care level to access. ^[56]^ Training was cited as a significant issue, with most respondents citing a lack of access to external training (80%). ^[56]^ Second, patients with a subtype of the HSDs will likely have challenges establishing a meaningful relationship with health care providers. A lack of familiarity with the concept of HSDs (still referred to as BJHS in electronic medical records), the debilitating effect of chronic pain, extra-articular manifestations that mimic a systemic autoimmune or inflammatory arthritis, and years of diagnostic delay make it challenging for a patient to place trust in health care providers when for months or years they have been diagnosed with other pain syndromes. Eleven patients with HSD/hEDS interviewed as part of a phenomenological hermeneutic study revealed that relationships among HSD/hEDS patients are adversely affected.^[57]^ These patients reported change in their social network and that various type of relationships were influenced by their disease.^[57]^

Lifestyle modification is potentially beneficial for patients with HSDs, although rigorous studies evaluating the efficacy of any particular intervention are lacking. Empiric strategies would include weight loss, incorporating anti-inflammatory foods and diet strategies (at least for overweight/obese patients, but perhaps for all), and participation in regular exercise.^[58]^ Modification or restriction of certain activities required by their employment might be needed stretching techniques should be focused on tight muscles, not lax joints.^[2,8]^ Neuromuscular taping and bracing may help to prevent injury and improve gait.^[59]^ Patients with HSDs should consider strengthening programs for periarticular musculature in order to stabilize joints.^[8,60]^ Some patients may be apprehensive of joint injury (or repeat joint injury), and for this cognitive behavioral therapy may be helpful.

Isometric exercises appear reasonable as they do not extend the muscle completely. An observational study of 27 female patients with HSDs/hEDS demonstrated isometric external rotation while holding a 2 kg dumbbell resulted in inferior translation whereas isometric shoulder flexion and shoulder/elbow extension respectively led to anterior-superior and superior translation.^[61]^ A randomized trial of 21 patients assessing multidirectional shoulder instability in HSD/hEDS patients found home-based exercise programs were beneficial for improving shoulder function using several different validated indices.^[62]^ Regarding intensity, some clinicians favor low-load strengthening exercises, concerned that high-load strengthening exercise may lead to joint injury, though this lacks support in the current literature. A randomized trial of 100 adult patients demonstrated that after 16 weeks of therapy, high-load shoulder strengthening exercise was statistically superior to low-load strengthening exercise for self-reported function.^[63]^ No serious adverse events were noted. A different study enrolled 14 HSD/ hEDS patients in a multidisciplinary rehabilitation regimen consisting of physical training to improve aerobic capacity, muscle strength and proprioception.^[64]^ At the end of 8 weeks patients showed a clinically relevant improvement in functional disability, muscle strength, perceived harmfulness, and pain intensity.

Pharmacologic treatment strategies are similar to that for other chronic pain conditions with avoidance of opioids and a deference toward non-steroidal anti-inflammatory drugs (NSAIDs)/acetaminophen, topical NSAIDs, and antidepressants (particularly serotonin and norepinephrine reuptake inhibitors (SNRIs) or low-dose tricyclic antidepressants (TCAs).^[2]^ Muscle pain or spasm may be treated with antispasticity agents such as baclofen or tizanidine, although patients with exercise intolerance or orthostatic symptoms may not tolerate TCAs or tizanidine. Patients with comorbid fibromyalgia may benefit from gabapentin or pregabalin.^[2]^ Patients surveyed through the Ehlers-Danlos Society diagnosed with EDS or HSD reported that the most commonly used therapies were NSAIDs, acetaminophen, opioids, and physical therapy (70%-92% of patients). Therapies rated as most efficacious were opioids, physical therapy, and marijuana.^[65]^ Patient-initiated complementary therapy use was widespread (56%) and mainly utilized by patients with higher reported levels of pain. Vitamin C may be useful in addressing some cutaneous features,^[3]^ and vitamin D supplementation may be required in those with reduced bone mineral density and hypovitaminosis D.^[65,66]^

## Conclusion

While the relationship between JH and pain has been known for decades, it remains a challenge identifying when JH is responsible for pain. Variability in patient symptoms and clinical presentation, clinician inexperience identifying pain attributable to hypermobility, and recent changes in nomenclature make diagnosing patients with a subtype of HSD fraught with difficulty. In addition to joint and regional pain attributable to muscle/tendon involvement, extra-articular manifestations are common. The current treatment of pain borrows from studies using older nomenclature as well as management of other chronic pain syndromes. A multidisciplinary approach is likely optimal, incorporating specialists such as physical therapist and mental health specialists to address the regional and chronic pain that patients with subtypes of HSD experience. Lifestyle modifications focused on weight loss and exercise create the foundation for therapeutic interventions for HSDs. Isometric exercises appear reasonably safe and effective but data is limited to regional management (shoulder). Pharmacologic therapies focus on judicious use of NSAIDs, acetaminophen, and use of SNRIs and TCAs for more widespread pain.
